# Essential role of Ahnak in adipocyte differentiation leading to the transcriptional regulation of Bmpr1α expression

**DOI:** 10.1038/s41419-018-0873-6

**Published:** 2018-08-28

**Authors:** Jong Kyu Woo, Jae Hoon Shin, Seo Hyun Lee, Hun-Min Park, Soo Young Cho, You Me Sung, Il Yong Kim, Je Kyung Seong

**Affiliations:** 1 Korea Mouse Phenotyping Center (KMPC), Seoul National University, Seoul, Republic of Korea; 2 Department of Surgery, University of Michigan, Ann Arbor, MI USA; 3 Laboratory of Developmental Biology and Genomics, College of Veterinary Medicine, Seoul National University, Seoul, Republic of Korea; 4 National Cancer Center, Goyang-si, Gyeonggi-do Republic of Korea; 5 Interdisciplinary Program for Bioinformatics, Program for Cancer Biology and BIO-MAX institute, Seoul National University, Seoul, 08826 Republic of Korea

## Abstract

The role of Ahnak in obesity has been reported previously. Loss of Ahnak leads to decreased Bmp4/Smad1 signaling, resulting in the downregulation of adipocyte differentiation. However, the biological significance of Ahnak remains largely unknown. In this study, we demonstrate that Ahnak-mediated impaired adipogenesis results in decreased Bmpr1α transcriptional expression. To confirm this, Ahnak siRNA was used to knock-down Ahnak in C3H10T1/2 and primary stromal vascular fraction cells. Ahnak siRNA transfected cells showed suppression of Bmpr1α expression and decreased BMP4/ Bmpr1α signaling. The differential adipogenesis was further confirmed by knock-down of Bmpr1α in C3H10T1/2 cells, which resulted in reduced adipogenesis. Moreover, stable Ahnak knock-out C3H10T1/2 cells stably transfected with Ahnak CRISPR/Cas9 plasmid suppressed expression of Bmpr1α and prevented differentiation into adipocytes. Furthermore, we developed immortalized pre-adipocytes from wild-type or Ahnak Knock-out mice’s stromal vascular fraction (SVF) to confirm the function of Ahnak in pre-adipocyte transition. Immortalized Ahnak knock-out SVF cells showed lower level of Bmpr1α expression, evidence by their impaired BMP4/Bmpr1α signaling. Upon adipogenic induction, immortalized Ahnak knock-out SVF cells exhibited a marked decrease in adipocyte differentiation compared with immortalized wild-type pre-adipocytes. Furthermore, over-expression of Bmpr1α restored the adipogenic activity of Ahnak knock-out C3H10T1/2 cells and immortalized Ahnak knock-out SVF cells. Our data reveal the missing link in Ahnak-mediated adipose tissue remodeling and suggest that precise regulation of Ahnak in adipose tissue might have a therapeutic advantage for metabolic disease treatment.

## Introduction

Obesity is characterized by the excessive adipocyte hypertrophy and hyperplasia caused by highly imbalance of energy expenditure vs. food intake^1^. Even though the clinical importance of obesity is growing, our understanding of the molecular mechanisms regulating the adipocyte differentiation response to adipose tissue expansion is extremely limited. Thus, interest is focused on adipocyte differentiation as a platform for anti-obesity therapeutic approaches^2^.

Adipose tissues play a major role in energy storage in mammals. Adipose tissues have been divided into two distinct types: white adipose tissues (WATs), which are the primary site of energy storage by accumulating lipids and brown adipose tissues (BATs)^3^. Compared to WAT, the principal function of BAT is thermogenic energy consumption. WAT primarily contributes to energy storage for the regulation of energy balance and serves as a central endocrine organ playing key roles in metabolism^4^. Adipose tissue is comprised of adipocytes, which are the majority of cells in adipose tissue. Adipocytes are critical in normal physiology. The dysfunction of adipocytes causes a diverse range of diseases, including obesity, diabetes, and lipodystrophies^5^, derived by adipogenesis from specific precursor cells. The pre-adipocyte to adipogenic differentiation (adipogenesis) is a key process in fat mass. Adipogenesis requires an orchestrated multi-step process of sequentially concerted signaling pathways for adipocyte formation^6,7^. The pre-adipocytes are derived from pluripotent mesenchymal stem cells (MSCs), which have the potential to commit to differentiate into adipocyte, myocyte, osteocyte, or chondrocyte lineages^8^, that become restricted to the adipocyte lineage through a multi-step procedure. Recruitment to this lineage gives rise to pre-adipocytes which undergo several stages and then differentiate into adipocytes^9^. The most critical transcription factors for adipogenesis are peroxisome proliferator-activated receptor gamma (Ppar-γ) and CCAAT enhancer-binding protein alpha (C/EBP-α). Early signaling events mediated by C/EBP-β and C/EBP-α contribute to initiate adipogenesis by inducing Ppar-γ, being both necessary and sufficient for white and brown adipocyte formation. In addition, they are also required to maintenance of the adipose tissues^7,10–12^.

Bone morphogenetic proteins (BMPs) were originally identified as factors that induce bone formation when implanted at ectopic sites^13^. Also, BMPs are transforming growth factor-beta (TGF-β) superfamily members that have pleiotropic functions during development by regulating embryogenesis, organogenesis, and morphogenesis^14,15^. They signal through a hetero-tetrameric complex of transmembrane receptors known as type I and II serine/threonine kinase receptors^16,17^. Seven type I receptor members have been identified, which are referred to as activin-like kinase (ALK1–ALK7), while three members of type II receptors are known (BMPR2, ACTR2, and ACTR2B). Both type I and type II receptors contain an N-terminal extracellular-binding domain, a single transmembrane region, and an intracellular serine/threonine kinase domain^16,18^. Binding of BMP to the BMP receptor complex induces a signaling cascade. The two types of BMP-induced signaling pathways include the canonical Smad-dependent signaling pathway and Smad independent signaling pathway, such as p38 mitogen-activated protein kinase (MAPK) pathways^18,19^. Recently, it was reported that adipocyte differentiation is closely linked to Bmpr1α/Smad1 signaling pathways^20,21^. BMP4/Bmpr1α expression is a key regulator for the commitment and differentiation of pluripotent C3H10T1/2 cells to the white adipocyte lineage^21,22^. We recently reported that mice with genetic ablation of Ahnak exhibited reduced white adipocytes through impairment of BMP4-mediated Smad signaling, which influences adipogenesis and glucose homeostasis^23^.

The neuroblast differentiation-associated protein, Ahnak, is an exceptionally large (700 kDa) and ubiquitously expressed essential protein for cell proliferation and migration^24–26^. Ahnak is expressed in several intracellular locations, including the plasma membrane, cytoplasm, and nucleus^27^. Ahnak is highly expressed in adipose tissue and is upregulated in high-fat diet-induced obesity models^28,29^. Our previous studies have reported that Ahnak is involved in obesity and cellular adipogenesis process^23,30^. However, the underlying molecular mechanism of Ahnak during adipogenesis is poorly understood.

We investigated whether Ahnak is involved in the programming of adipogenesis in pre-adipocytes. In the current study, we verified that Ahnak can control adipogenic differentiation by down-regulating Bmpr1α expression in pre-adipocytes. The proposed inhibitory effect of Ahnak knock-out on white adipogenesis prompted us to further investigate the involvement of Bmpr1α in adipocyte differentiation. Bmpr1α over-expression restored Ahnak-mediated impairment of adipogenesis in pre-adipocytes. These data implicate Ahnak as a new regulatory factor that maintains proper adipogenic differentiation. Our findings reveal the mechanism by which Ahnak regulates adipocyte differentiation.

## Results

### Deficiency of ahnak downregulates bmpr1α expression in mouse adipose tissues

**Fig. 1:**
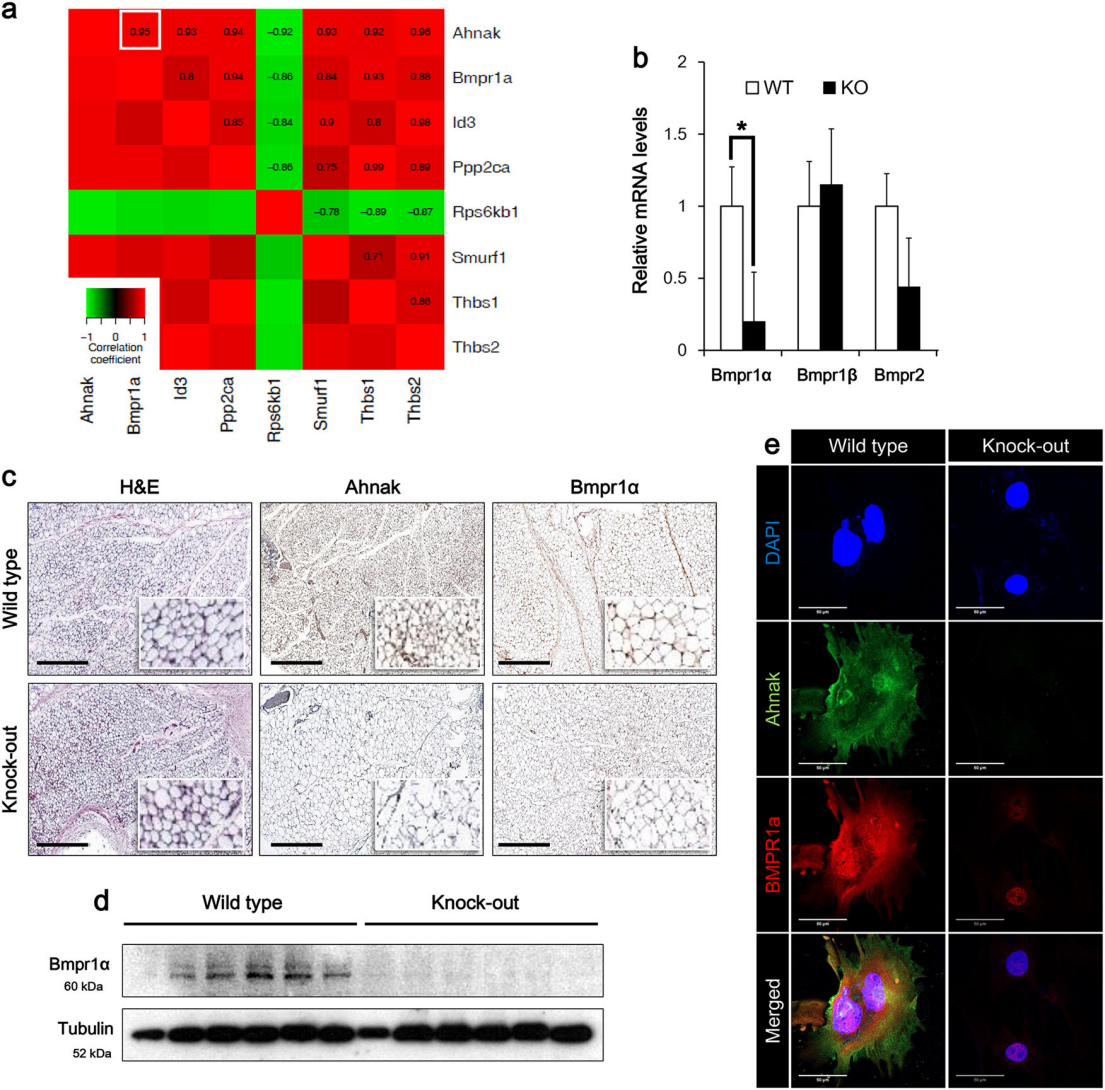
Ahnak knock-out suppresses Bmpr1α expression. **a** The transcriptome profile from obesity and diabetes by blockade of TGF-β/Smad3 signaling. The colors ranging from green to red represent Pearson correlation, indicating low to high correlations, respectively. **b** Relative expression of Bmpr1α was determined by qRT-PCR analysis (right panel). GAPDH serves as a loading control. Wild type (WT) and Ahnak knock-out (KO). **c** Immunohistochemical analysis of Ahnak or Bmpr1α in mouse inguinal white adipose tissues (iWAT). **d** Western blotting analysis of lysates harvested from wild-type and Ahnak Knock-out mice iWAT. **e** Immunofluorescence staining of Ahnak or Bmpr1α in primary SVF isolated from wild-type or Ahnak knock-out mouse iWAT

Previously, we reported the role of Ahnak in obesity and energy expenditure in mice model^23^. Deficiency of Ahnak leads to decreased amount of adipose tissues and resistance to obesity by the impairment of Smad signaling pathways. To address the molecular mechanism of Ahnak-mediated BMP4/Smad1 signaling impairment, we used microarray data blocked TGF-b/Smad3 signaling in WAT of Smad3-deficient mice (GSE28598). Yadav et al. constructed a transcriptome profile for obesity and diabetes by blockade of TGF-β/Smad3 signaling in WAT of Smad3-deficient mice^31^. First, we selected 82 TGF-β signaling pathway-related genes from the KEGG database. The co-expression patterns between Ahnak and the TGF-β signaling pathway gene sets were identified by Pearson correlation (*p* value < 0.05) and seven genes were selected. Several genes known to participate in the adipogenic process were correlated with Ahnak expression, including Bmpr1α, Id3, Ppp2ca, Smurf1, Thbs1, and Thbs2 (Fig. 1a). We found that the most correlatively expressed gene was Bmpr1α. We evaluated Bmpr1α expression in iWAT. As shown in Fig. 1b, Bmpr1α mRNA expression was reduced in iWAT from Ahnak knock-out (KO) mice compared to wild-type (WT) mice. In contrast, minor differences in Bmpr1β and Bmpr2 expression occurred in Ahnak KO mice. Furthermore, histological analysis revealed that Ahnak KO iWAT showed decreased expression of Bmpr1α (Fig. 1c). Furthermore, Bmpr1α protein levels are also measured by western blotting using mice iWAT (Fig. 1d). We confirmed that the Bmpr1α expression was dependent on Ahnak expression in vivo. But, we could not detect Ahnak protein band since Ahnak protein size are larger than 700 kDa. Ahnak western blotting always showed poor band. Then, we performed indirect immunofluorescence (IF) analysis to examine the Ahnak and Bmpr1α proteins expression in primary stromal vascular fraction (SVF), that were isolated from Ahnak knock-out or WT mice iWAT, and Ahnak and Bmpr1α proteins were visualized by confocal microscope. Bmpr1α expression was predominantly decreased in Ahnak knock-out SVF compared with WT SVF (Fig. 1e). Altogether, these results identified Ahnak as a marker expressed in SVF, with a positive correlation between Bmpr1α mRNA and protein expression.

### Ahnak knock-down in C3H10T1/2 cell or primary SVF suppresses bmpr1α expression and BMP4/smad1/5 signaling pathway

**Fig. 2:**
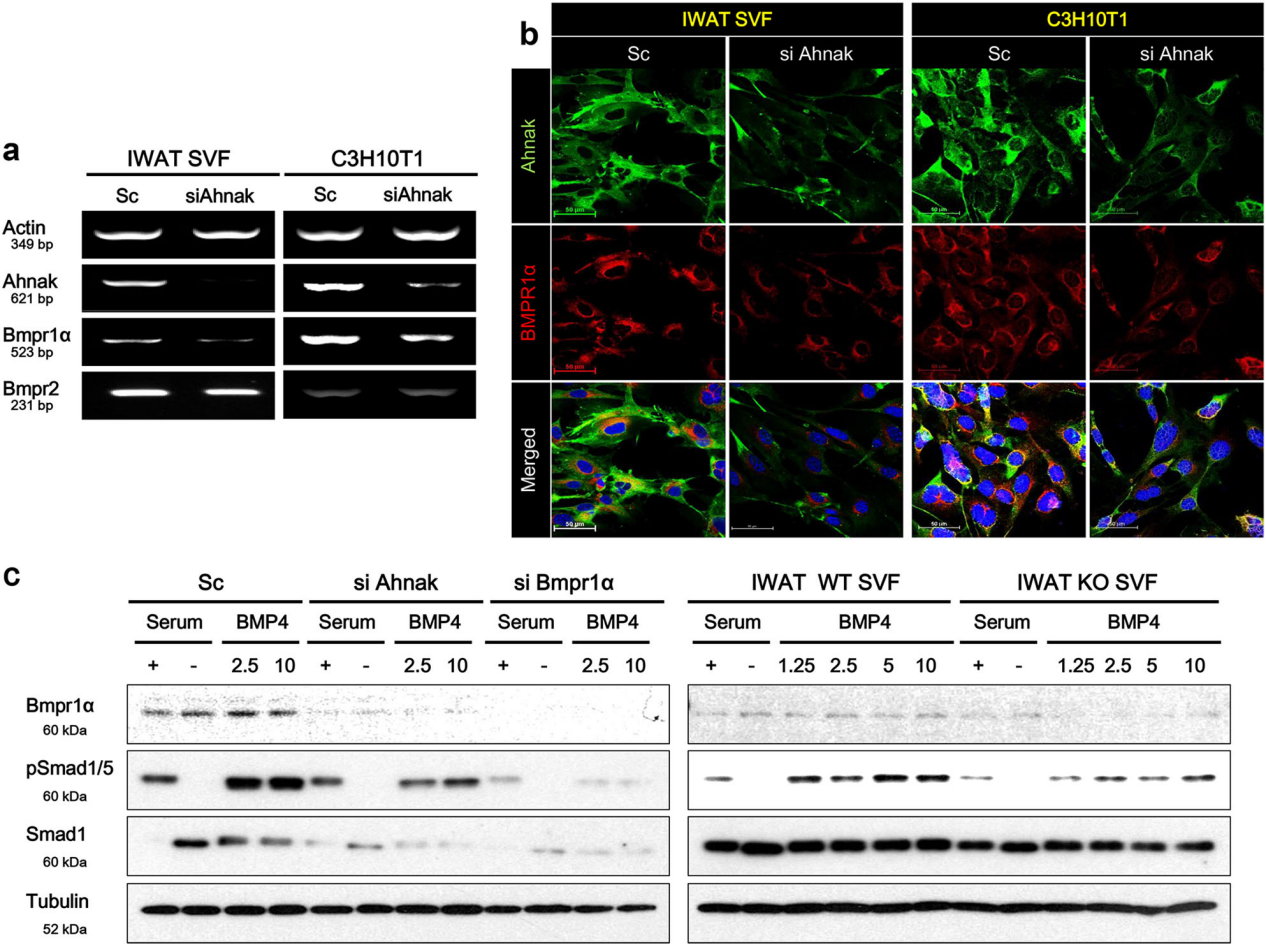
Ahnak knock-down inhibits Bmpr1α expression and BMP4-mediated Smad1/5 activation. Primary mouse iWAT SVF and C3H10T1/2 cells were transfected with siRNA targeting for Ahnak. Ahnak or Bmpr1α levels were determined by semi-quantitative RT-PCR analysis (**a**) and Ahnak and Bmpr1α immunofluorescence staining (**b**). **c** To assess the downstream signaling cascade of BMP4 on Ahnak SiRNA transfection, Ahnak or Bmpr1α SiRNA transfected cells were acutely stimulated with recombinant mouse BMP4 for 2 h and analyzed by western blotting for phosphorylated Smad1/5. Results are normalized with tubulin

To confirm whether Ahnak expression directly modulates Bmpr1α expression, we transfected Ahnak siRNA into C3H10T1/2 cells or primary SVF cells, and tested the level of Bmpr1α by RT-PCR and IF analysis. As Fig. 2a shows, Ahnak mRNA expression was downregulated in primary iWAT SVF and C3H10T1/2 cells by Ahnak siRNA. Ahnak depletion contributed to the downregulation of Bmpr1α mRNA expression. However, Bmpr2 mRNA expression was not changed by Ahnak knock-down in two cell lines. It suggests that Ahnak may regulate Bmpr1α specifically. The same results were observed in IF analysis. Primary iWAT SVF and C3H10T1/2 cells showed similarly reduced Bmpr1α expression with Ahnak siRNA transfection (Fig. 2b). Thus, we suggest that the Ahnak and Bmpr1α expression might be linked in pre-adipocytes and participate in adipocyte maintenance or differentiation. Phosphorylation of Smad1/5 by the BMP4/Bmpr1α signaling cascade regulates target gene transcription within the nucleus. Western blotting of whole cell lysate after 4 h of stimulation with BMP4 (2.5 or 10 ng/mL) confirmed the lack of induction of Smad1/5 phosphorylation in Ahnak or Bmpr1α deficiency cells. As shown in Fig. 2c (left panel), Ahnak siRNA or Bmpr1α siRNA transfection resulted in an abrogation of BMP4-induced Smad1/5 phosphorylation in C3H10T1/2 cells. We next determined a Smad1/5 phosphorylation level in primary SVF between wild-type (WT) or Ahnak knock-out (KO) mice. SVF were collected from mice iWAT, cultured until reaching 80–90% confluence, and the cells were cultured in serum-free medium for 24 h, then stimulated with sequential doses of BMP4 for 4 h prior to western blotting analysis. Treatment of primary WT SVF with BMP4 resulted in phosphorylation of Smad1/5 but in Ahnak knock-out SVF, we observed reduced BMP4-stimulated Smad1/5 phosphorylation (Fig. 2c, right panel). This suggests the downregulation of Ahnak in pre-adipocytes suppresses Bmpr1α-mediating BMP4 signaling cascade that might have a pivotal role in the process of adipogenesis.

### Knock-down of ahnak or bmpr1α impairs adipogenic differentiation of C3H10T1/2 cells

**Fig. 3:**
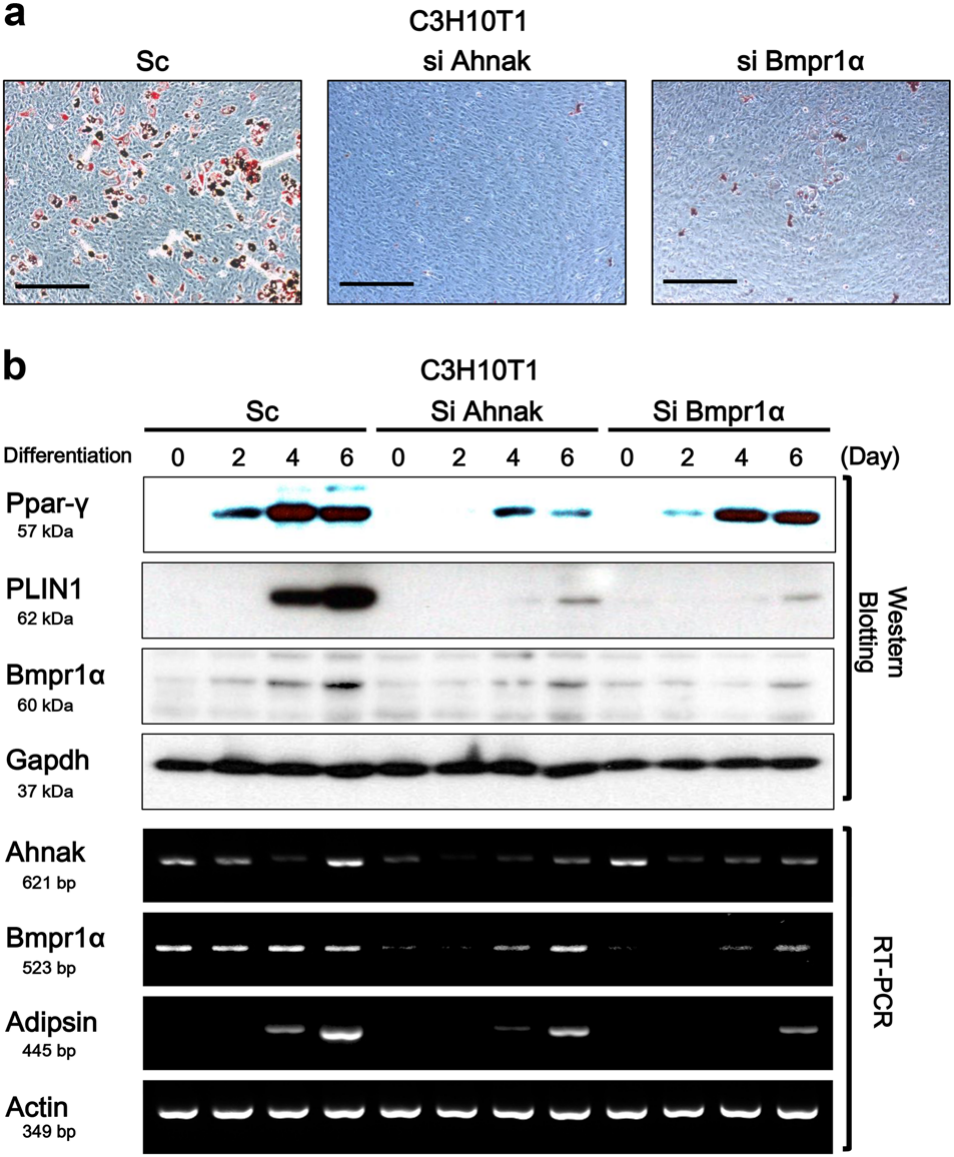
Ahnak knock-down inhibits white adipocyte differentiation. C3H10T1/2 cells were transfected with siRNA targeting Ahnak or Bmpr1α, and cells were assessed for standard white adipocyte differentiation. **a** Adipogenic differentiation was determined using Oil Red O staining. **b** Protein or mRNA level of white adipogenic markers were analyzed by western blotting and semi-quantitative RT-PCR

Phosphorylated Smad1/5 are intermediates in the BMP4/Bmpr1α signaling pathways of various cell types^18,19^. To investigate the effect of Ahnak-mediated Bmpr1α suppression on adipogenic differentiation of C3H10T1/2 cells, we knock-downed Ahnak or Bmpr1α in C3H10T1/2 and compared their differentiation capacity with scrambled siRNA transfected cells. Cells at 80–90% confluence were transfected with Ahnak or Bmpr1α siRNA and were treated to promote differentiation to white adipocytes. Ahnak or Bmpr1α was required for lipid storage in differentiating C3H10T1/2 cells. Oil Red O staining revealed that knock-down of Ahnak or Bmpr1α in the C3H10T1/2 cells displayed significant suppression of their differentiation capacity (Fig. 3a). Expression of adipogenic protein markers, such as peroxisome proliferator-activated Pparγ and perilipin-1 (PLIN1), was significantly decreased in Ahnak or Bmpr1α downregulated cells, as compared with scrambled controls (Fig. 3b, upper panels). Furthermore, mRNA levels also confirmed Ahnak-mediated or Bmpr1α-mediated impairment of adipogenesis (Fig. 3b, lower panels). Interestingly, we confirmed that the Bmpr1α expression was dependent on Ahnak expression in C3H10T1/2 cells. The positive correlation between Ahnak and Bmpr1α expression was seen only in Ahnak SiRNA transfection, but Bmpr1α siRNA transfection did not alter Ahnak expression. Considering that Ahnak deficiency is likely to attenuate adipogenesis by suppression of Bmpr1α expression, however, the behind mechanism of Ahnak deficiency mediates Bmpr1α suppression in pre-adipocytes remains to be further investigated.

### Ahnak gene disruption with CRISPR/Cas9 on C3H10T1/2 cells suppressed BMP4/smad1/5 signaling pathways and white adipocyte differentiation

**Fig. 4:**
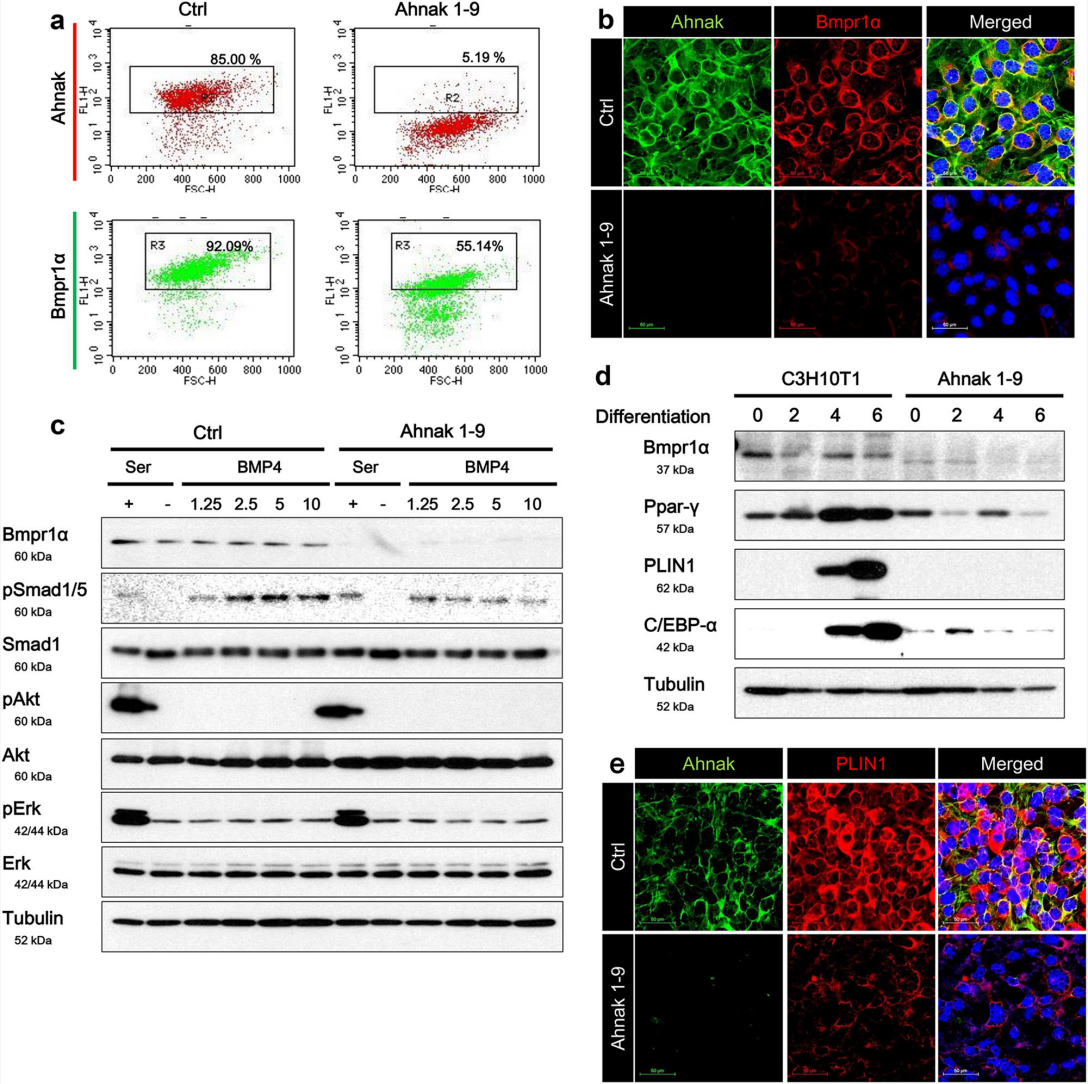
Stable Ahnak knock-out suppresses Bmpr1α expression and white adipogenesis. Deleting Ahnak by CRISPR/Cas9 genome editing in C3H10T1/2 cells. C3H10T1/2 cells were transfected with control and Ahnak CRISPR/Cas9 plasmids, respectively, and then subjected to clonal selection. Ahnak and Bmpr1α expression were determined in Ahnak knock-out cells by FACS analysis (**a**) and immunofluorescence staining (**b**). **c** Western blotting analysis of lysates collected from recombinant mouse BMP4-stimulated Ahnak knock-out C3H10T/2 cells (Ahnak 1–9). The cells were treated with 1.25, 2.5, 5, and 10 ng/mL of BMP4 for 2 h, and the analyzed by indicated antibodies. Ahnak 1–9 cells were subjected to white adipocyte differentiation procedure. White adipogenic differentiation was estimated by western blotting with Ppar-γ, PLIN1, and C/EBP-α (**d**) and immunofluorescence analysis with Ahnak and PLIN1 double staining. Nuclei were stained with DAPI (**e**)

To corroborate the effect that Ahnak exerts on white adipogenic differentiation, we used CRISPR/Cas9 gene disruption^32^ to disrupt Ahnak using a single guide RNA approach in the C3H10T1/2 cells. From stable Ahnak CRISPR/Cas9 knock-out cell lines, termed Ahnak 1–9, we found that adipogenesis was significantly abolished (Fig. 4e) by Ahnak gene disruption, and was associated with loss of Bmpr1α protein (Fig. 4a). After selection of Ahnak knock-out cells, Ahnak 1–9, demonstrated ~45% reduction of Bmpr1α proteins expression as shown by FACS analysis. In addition, immunofluorescence analysis showed Bmpr1α downregulation in Ahnak 1–9 cells (Fig. 4b). Given the critical roles of Bmpr1α in adipogenesis and Smad1/5 signaling cascades, we examined whether Smad1/5 phosphorylation was inhibited by Ahnak gene deletion. As shown in Fig. 4c, Smad1/5 was activated by presence of BMP4 in C3H10T1/2 control cells, but BMP4-mediated Smad1/5 phosphorylation was blocked by Ahnak disruption in Ahnak 1–9 cells. Ahnak gene disruption also inhibited induction of Ppar-γ and C/EBPα in white adipogenesis stimulation (Fig. 4d). In addition, Ahnak 1–9 cells displayed decreased fat accumulation when white adipocyte differentiation was induced compared with the C3H10T1/2 control cells as demonstrated by reduced PLIN 1 immunofluorescence staining (Fig. 4e). Altogether, these results indicated that Ahnak gene disruption subsequently downregulated the expression of Bmpr1α, together with BMP4-mediated Smad1/5 activation, thus inhibiting the adipogenic differentiation of C3H10T1/2 cells.

### Immortalized iWAT SVF cells showed different adipogenic differentiation capacity

**Fig. 5:**
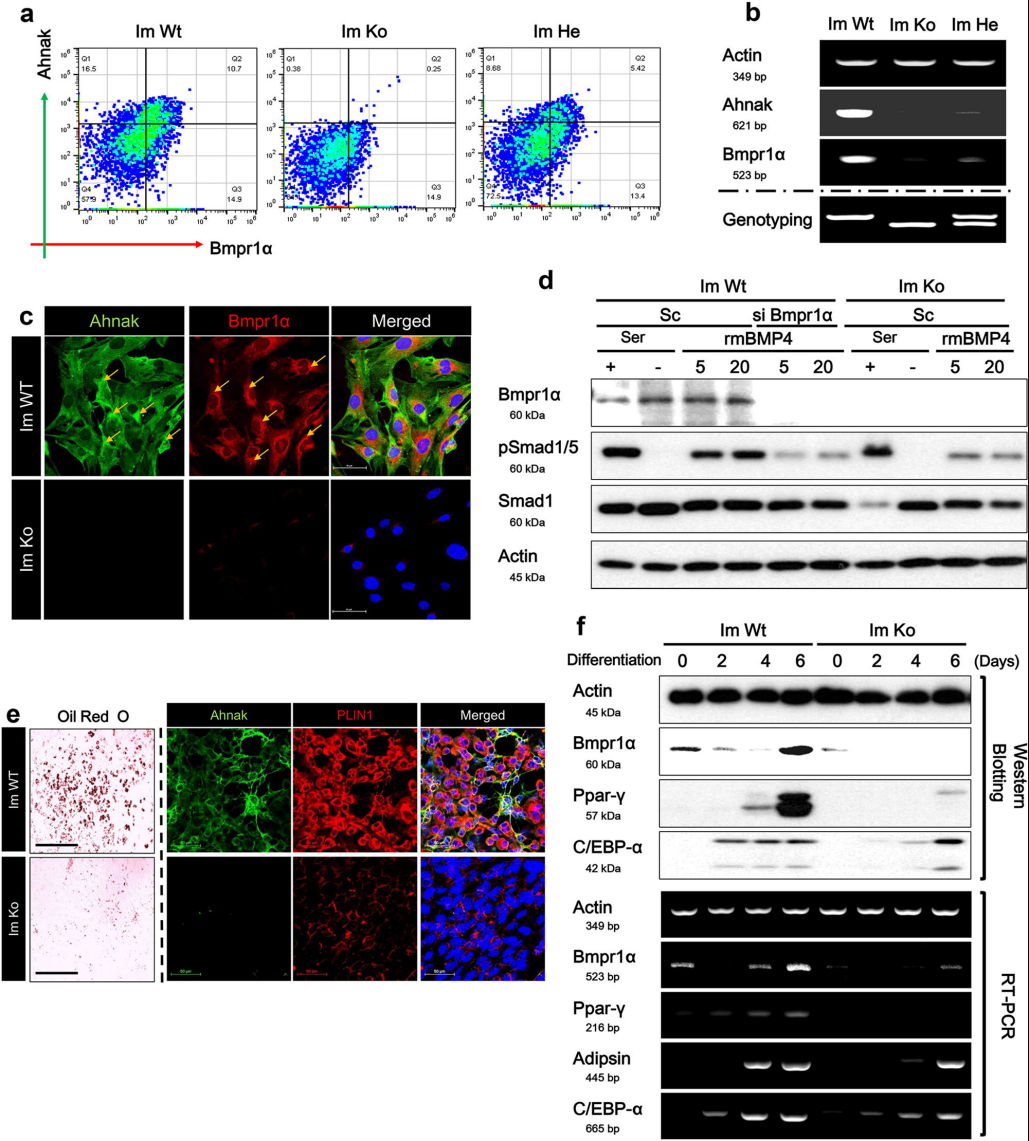
Immortalized Ahnak knock-out SVF cells show Bmpr1α suppression and impairment of adipogenesis. Mouse iWAT SVF cells were immortalized by SV40-LT antigen introduction and induced to white adipocyte differentiation. **a** Tubulin serves as a loading control. Ahnak and Bmpr1α expression were determined in Immortalized Knock-out cells (Im Ko) by FACS analysis (**a**), semi-quantitative RT-PCR analysis (**b**) and immunofluorescence staining (**c**). **d** Phosphorylated Smad1/5 level was measured by western blotting analysis in Im Ko cells treated with indicated concentration of BMP4 (ng/mL) for 2 h. Im Ko cells were induced to white adipogenic differentiation that was determined by immunofluorescence staining (**e**) and protein or mRNA level of white adipogenic markers was analyzed by western blotting analysis and semi-quantitative RT-PCR analysis (**f**)

**Fig. 6:**
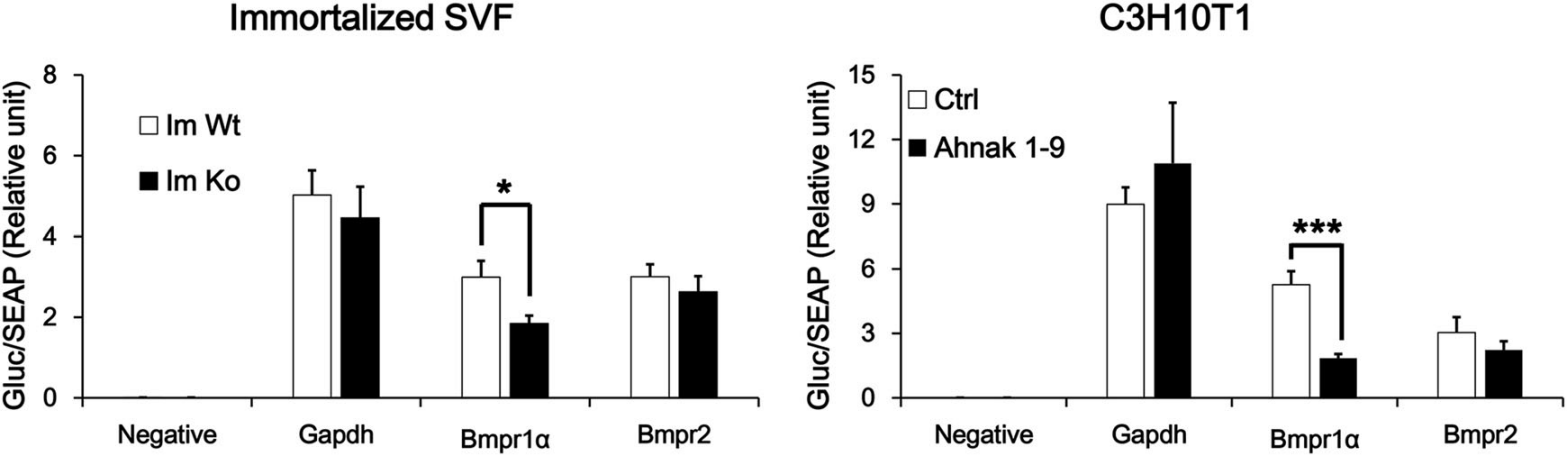
Ahnak regulates Bmpr1α transcriptional activity. The immortalized Ahnak SVF (left panel) and Stable Ahnak knock-out (right panel) and cells were transfected with indicated plasmid encoding mouse GAPDH promoter region (−1333/+13), Bmpr1α promoter region (−1012/+366), and Bmpr2 promoter region (−1492/+179) with gaussia luciferase construct. Cells were transfected for 2 days and supernatant were collected for the luciferase assays. Luciferase activity was normalized to secreted alkaline phosphatase. Data are presented as the mean ± s.d. ** or *** indicates a *p* value 0.01 or 0.001 by Student’s *t*-test analysis

To obtain unbiased insight into putative Ahnak-dependent molecular pathways impacting Bmpr1α-mediated adipocyte differentiation, we developed immortalized pre-adipocytes derived from SVF of WT, Ahnak hetero zygote (He), and Ahnak knock-out mice iWAT (Im WT, Im He, and Im Ko, respectively). The immortalized cells were passaged in culture for more than 30 days and were followed for up to 10 passages. The induction of adipocyte differentiation was tested through the use of a standard protocol for white adipogenesis that employs a cocktail of insulin, dexamethasone, and 3-isobutyl-1-methylxanthine (IBMX). Having established that immortalized cells were capable of differentiating into adipocytes in vitro and that the immortalized cells retained differentiation characteristics of primary cells, we performed fluorescent-activated cell sorting (FACS) analysis to verify Ahnak and Bmpr1α expression in immortalized cells. FACS analysis revealed that 10.7% of cells expressed Ahnak and Bmpr1α together in Im WT cells. The Ahnak/Bmpr1α double-positive population comprised approximately half of the cells population (5.42%) present in the Im He cells compared as Im WT cells, but Im Ko cell was negative for Ahnak and Bmpr1α double-positive population. Indeed, Ahnak negative Bmpr1α-positive populations were similar among all three cell lines (Fig. 5a). These results unambiguously demonstrate the role of Ahnak protein, which controls Bmpr1α expression in SVF cells. Bmpr1α mRNA expression was further confirmed by RT-PCR. Bmpr1α downregulation in Im He and Im Ko cells provided evidence of the regulatory role of Ahnak (Fig. 5b). Additionally, immortalized SVFs were immunofluorescently stained by a specific antibody for Ahnak and Bmpr1α and were analyzed using confocal laser microscopy (Fig. 5c). As expected, Im WT cells retained an identical staining pattern of Ahnak and Bmpr1α. In contrast, the Im Ko cells showed a poor staining pattern of Ahnak and Bmpr1α under basal conditions. Consistent with FACS and mRNA expression data, Bmpr1α protein was decreased in Im Ko cells by IF analysis. Western blotting with the antibodies that recognizes the Bmpr1α and phosphorylated Smad1/5 revealed a robust decrease in BMP4-stimulated phosphorylation in Im Ko cells compared as Im WT cells. Nonetheless, BMP4-stimulated phosphorylation of Smad1/5 was almost abolished in Im Ko cells with no change in total Smad1 protein or the Actin protein (Fig. 5d). To confirm the role of Bmpr1α on BMP4-stimulated phosphorylation of Smad1/5, Im WT cells were transfected with Bmpr1α SiRNA and then stimulated with BMP4. BMP4-stimulated phosphorylation of Smad1/5 was also significantly decreased with no change in Smad1 protein (Fig. 5d). SVF cells of adipose tissue are characterized by their capacity to differentiate into mature adipocytes^33^. The immortalized SVF cells were subjected to adipogenesis. When the accumulation of intracellular lipids was confirmed by Oil Red O staining, the staining intensity was decreased in Im Ko cells. In addition, PLIN1 staining was also decreased in Im Ko cells during adipogenesis (Fig. 5e). Expression of adipogenic markers, such as Ppar-γ and C/EBPα proteins and mRNA, were significantly decreased in Im Ko cells compared with Im WT (Fig. 5f). These results revealed an apparent decrease in the expression of Bmpr1α in Ahnak 1–9 and Im Ko cells (Figs. 4 and 5). These results indicate that Ahnak deficiency-mediated suppression of Bmpr1α occurred at the transcriptional level through the downregulation of transcriptional activity. As showed in Fig. 6a, b, Ahnak deficiency directly targeted Bmpr1α promotor transcriptional activity. Taken together, the results unambiguously demonstrate the necessity of Ahnak in white adipogenesis whether upregulated Bmpr1α expression for pre-adipocytes differentiation by BMP4/Smad1/5 signaling cascade.

### Over expression of bmpr1α restores ahnak deficiency-mediated adipogenesis impairment

**Fig. 7:**
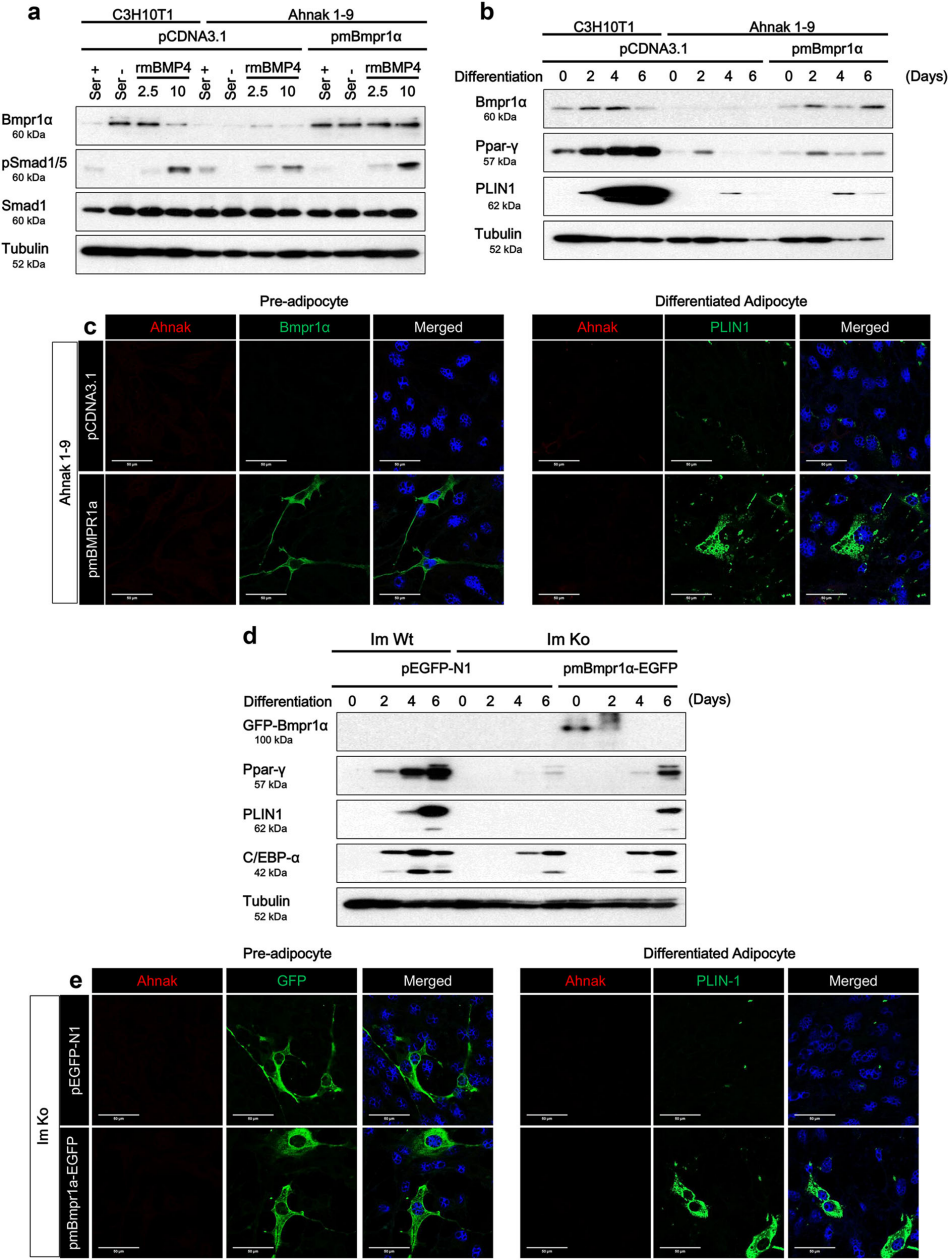
Bmpr1α over-expression induces white adipogenesis in Ahnak knock cells. Ahnak knock-out C3H10T1/2 (Ahnak 1–9) cells were transfected with mouse Bmpr1α encoded plasmid. Transfected cells were stimulated with indicated concentration of recombinant mouse BMP4 (ng/mL) for 2 h. (**a**) Western blotting analysis was performed with indicated antibodies. Tubulin was used as an internal control. After transfection, white adipocyte differentiation was induced. The effect of Bmpr1α on adipogenesis in Ahnak deficiency cells were assessed by western blotting analysis (**b**) and immunofluorescence analysis with Ahnak and PLIN1 double staining. Nuclei were stained with DAPI (**c**). Immortalized Ahnak knock-out (Im Ko) cells were subjected upon Bmpr1α over-expression. The effect of Bmpr1α conjugated eGFP on adipogenesis were determined by western blotting analysis (**d**) and immunofluorescence analysis with Ahnak and PLIN1 double staining. Nuclei were stained with DAPI (**e**)

To assess functional involvement of Bmpr1α in Ahnak deficiency-mediated adipogenesis impairment, we applied ectopic mouse Bmpr1α expression in Ahnak knock-out C3H10T1/2 cells (Ahnak 1–9) and Immortalized Ahnak knock-out SVF cells (Im Ko). Western blotting analysis demonstrated a clear increase of Bmpr1α expression in the Bmpr1α introduced Ahnak 1–9 cells. Then, we determined the functional effects of ectopic expressed Bmpr1α by analyzing the downstream signaling upon BMP 4 stimulation. The Bmpr1α introduced Ahnak 1–9 cells showed increased phosphorylation of the Smad1/5 by BMP 4 stimulation (Fig. 7a). Furthermore, IF staining of these Bmpr1α transfected Ahnak 1–9 cells showed an intense Bmpr1α staining in contrast to the control vector transfected Ahnak 1–9 cells (Fig. 7c, left panel). Thus, ectopic Bmpr1α over-expression repaired the impaired adipocyte differentiation caused by Ahnak deficiency as assessed by the expression of the adipogenesis specific marker proteins Ppar-γ and PLIN1 (Fig. 7b). These findings were confirmed by IF analysis (Fig. 7c, right panels); PNL1 expression was much higher in Bmpr1α transfected Ahnak 1–9 cells. In addition, we examined whether Bmpr1α also contributes to adipogenic impairment in Im Ko cells. The cells were transiently transfected with eGFP-conjugated mouse Bmpr1α. As expected, the levels of Ppar-γ, PLIN1, and C/EBP1α were increased in Im Ko cells (Fig. 7d, e). Thus, Bmpr1α expression overcomes Ahnak deficiency related adipogenesis impairment in pre-adipocytes. The results corroborated the functional role of Ahnak during adipogenic differentiation mediated through the Bmpr1α signaling pathway.

## Discussion

Ahnak plays a pivotal role in adipocyte differentiation. We previously found that Ahnak functions in obesity resistance and insulin sensitivity by regulation of Smad1/5 signaling pathways. Genetic depletion of Ahnak protects from obesity and enhances insulin sensitivity^23^. Here, we show that Ahnak deficiency in pre-adipocytes negatively regulates Bmpr1α expression and adipogenic differentiation. Adipogenesis is a step-wise process. Pre-adipocyte first undergo growth arrest, re-enter the cell cycle under the influence of differentiation inducers, undergo mitotic clonal expansion and, finally, exit the cell cycle to undergo terminal differentiation^9,34^. BMP/Smad signaling pathways play pivotal roles in terminal differentiation from the pre-adipocytes into adipocytes^20,22^. In this research, we discovered that Bmpr1α, the adipogenesis regulator, was significantly linked with Ahnak in mouse adipose tissues. Emerging evidence showed that Ahnak knock-out inhibits the transcriptional activity of Bmpr1α in adipose tissues. As shown in Fig. 1b, the mRNA level of Bmpr1α was decreased in iWAT of Ahnak knock-out mouse. We also showed decreased Bmpr1α expression in SVF (Fig. 1d). Then, we showed that transient Ahnak knock-down suppresses Bmpr1α expression and BMP4-dependent Smad1/5 phosphorylation (Fig. 2). A previous study demonstrated that loss of Ahnak suppresses Smad1/5 phosphorylation. Our results support the view that Ahnak-mediated Bmpr1α suppression regulates adipogenesis by modulating BMP4-dependent Smad1/5 signaling. Our data demonstrate that Ahnak is required for maintaining of Bmpr1α expression for pre-adipocyte differentiation.

Ahnak knock-down inhibited differentiation of C3H10T1/2 cells to white adipocytes as measured by Ppar-γ and PLIN1 levels. In support of our findings, knock-down of Bmpr1α also impaired white adipogenesis. Of note, Ahnak deficiency-mediated adipogenesis inhibition was independent from altered cell viability (data not shown). Thus, Ahnak protein acts as a regulator that promotes the differentiation of pre-adipocytes to white adipocytes via Bmpr1α expression. Although it has been reported that Ahnak knock-out mice display obesity resistance and elevated energy expenditure, the underlying molecular mechanism is unclear^23^. In this study, we reveal the molecular links between Ahnak and Bmpr1α in relation to white adipogenesis.

Establishment of stable transfected C3H10T1/2 cells with Ahnak CRIPSR/Cas9 plasmid prevented differentiation into white adipocytes. The cells remained spindle shaped, while control cells became white adipocytes, indicated by Ppar-γ, C/EBP-α, and PLIN1 measurements. Furthermore, we developed immortalized Ahnak knock-out SVF cells and subjected them to standard white adipogenic differentiation process in vitro. Ahnak knock-out SVF cells showed consistent and significantly decreased BMP4/Smad1/5 signaling pathways and white adipogenic differentiation. The differential white adipogenesis was further confirmed by IF staining and decreased Ppar-γ, C/EBP-α, and PLIN1 expression. These data demonstrate that Ahnak negatively regulates white adipogenic differentiation of pre-adipocytes.

Thereafter, we investigated whether over-expression of Bmpr1α recovered Ahnak deficiency effect in Ahnak knock-out pre-adipocytes. We examined the stable Ahnak knock-out C3H10T1/2 cells (Ahnak 1–9) or immortalized Ahnak knock-out SVF cells (Im Ko). Mouse Bmpr1α over-expressing cells showed enhancement of the white adipogenesis compared with mock control cells. White adipogenic marker proteins Ppar-γ, C/EBP-α, and PLIN1 were significantly increased, and BMP4-dependent Smad1/5 activation was enhanced (Fig. 7). In addition, accumulation of intracellular lipid droplets were increased in Bmpr1α over-expressing Ahnak knock-out cells, as shown by PLIN1 IF staining.

Ahnak deficiency suppresses the expression of Bmpr1α and the BMP4/Smad1/5 signaling pathway to initiate adipogenic differentiation. Our findings demonstrate that: (1) Ahnak knock-out mice adipose tissues display decreased Bmpr1α transcriptional activity; (2) knock-out of Ahnak inhibits Bmpr1α expression and adipogenesis in C3H10T1/2 cells; (3) blockage of Smad1/5 signaling pathways by knock-out of Ahnak in C3H10T1/2 or SVF induced by BMP4; (4) knock-out of Ahnak prevents adipogenic differentiation in C3H10T1/2 or SVF cells; and (5) gain-of-function experiments showed that Bmpr1α expression recovered white adipogenesis in Ahnak knock-out pre-adipocyte cells. Upon BMP4 stimulation, Bmpr1α becomes activated and phosphorylates regulatory Smad1/5. The activated Smad1/5 is then translocated to the nucleus and binds to target gene promoters to regulate transcription of various adipogenesis regulating genes.

As Ahnak deficiency mediates obesity resistance, we proposed possible mechanism that Ahnak cooperates with Bmpr1α, which regulates the BMP4/Smad1/5 signal transduction. Taken together, our data provide evidence that Ahnak proteins in the adipose tissues control adipocyte differentiation functions through Bmpr1α-mediated BMP4/Smad1/5 signaling pathways. These findings may have therapeutic implications for the treatment of obesity or related metabolic diseases, such as cachexia and lipodystrophy.

## Materials and methods

### Antibodies and materials

Antibodies to β-actin, tubulin, glyceraldehyde-3-phosphate dehydrogenase (GAPDH), and enhanced green fluorescent protein (eGFP) and Ahnak CRISPR/Cas9 plasmid were purchased from Santa Cruz Biotechnology (Santa Cruz, CA, USA). Anti-Ahnak and Bmpr1α antibodies were obtained from Abcam (Cambridge, UK). Anti-Ppar-γ, perilipin-1 (PLIN1), phosphorylated Smad1/5 (pSmad1/5), Smad1, phosphorylated AKT (pAKT), AKT, phosphorylated extracellular signal-regulated kinase (ERK)1/2 (pERK1/2), ERK1/2 and C/EBPα antibodies were purchased from Cell Signaling Technology (Beverly, MA, USA). 3-Isobutyl-1-methylxanthine (IBMX) and triiodothyronine (T3) were obtained from Cayman Chemical (Ann Arbor, MI, USA), Dexamethasone (DEX), rosiglitazone (Rosi), insulin, and 0.5% Oil Red O were purchased from Sigma-Aldrich (St. Louis, MO, USA). Fetal bovine serum, Dulbecco’s phosphate buffered saline (DPBS), Dulbecco’s modified Eagle’s medium (DMEM), and DMEM/F12 were purchased from WelGene (Daegu, Korea). Secrete-Pair Dual Luminescence Assay Kit and pEZX-Bmpr1α promoter plasmid were obtained from GeneCopoeia (Rockville, MD, USA)

### Stromal vascular fraction cells isolation and immortalization

SVF was isolated from inguinal white adipose tissue (iWAT) of 6-week-old male Wild-type or Ahnak Knock-out mice^35^. Briefly, the adipose tissue was digested with 0.075% collagenase type II (Worthington Biochemical Corporation, Lakewood, NJ, USA) containing 1% bovine serum albumin (BSA) for 2 h at 37 °C with shaking. Following complete digestion, the mature adipocytes were separated from the SVF by centrifugation at 1200 g for 10 min. The floating mature adipocytes were discarded, and the precipitated SVF cells were re-suspended with 1× red blood cell (RBC) lysis buffer (eBioscience, ThermoFisher, Waltham, MA, USA) for 10 min at room temperature. After RBC lysis, SVF were passed through 40 μm cell strainers (SPL Life Sciences, Pocheon, Gyeonggi, Korea) and the cells were washed twice with DPBS. Isolated SVF cells were cultured in DMEM/F12 containing 10% FBS and antibiotics. For immortalization, cells at 40–60% confluency were transfected with SV40 LT/pSG5 viral construction, and 5 μg/ml puromycin was added to select the stable cells. All mice were maintained under specific pathogen-free conditions in the institutional animal facility of the College of Veterinary Medicine, Seoul National University. The experiments were performed according to the “Guide for Animal Experiments” (Edited by Korean Academy of Medical Sciences) and approved by the Institutional Animal Care and Use Committee (IACUC) of the Seoul National University (Permit Number: SNU-130903-1).

### In vitro adipogenesis and oil red O staining

C3H10T1/2 cells and immortalized pre-adipocytes were grown to confluency for 2 days and were cultured in white induction medium containing the 5 μg/mL insulin, 1 μM DEX, 0.5 μM IBMX, and with or without 0.5 μM Rosi. After 2 days, cells were maintained in DMEM/F12 supplemented with 10% FBS, 5 μg/mL insulin, and 1 nM T3 until the end of the experiments. After differentiation, the adipocytes were visualized by Oil Red O staining. Cells were washed three times with PBS and fixed for 15 min with 4% paraformaldehyde. Oil Red O solution (0.5%) freshly diluted into distilled water at a ratio of 3:2 was incubated with the fixed cells for 1 h at room temperature. Cells were washed with water and the stained lipid droplets in the adipocytes were visualized by light microscopy and photographed.

### siRNA, plasmid and transfection

All transfection was performed using the TransIT-Xs Dynamic delivery system (Mirus, Madison, WI, USA) following the manufacturer’s protocol. Transient knock-down of Ahnak and Bmpr1α was performed in primary SVF and C3H10T1/2 cells. Pre-designed Ahnak, Bmpr1α and control scrambled siRNA were purchased from BIONEER (Daejeon, Korea). Cells were transfected with 50 μM siRNA. Full-length mouse complementary DNA sequence for Bmpr1α was obtained from GeneCopoeia and subsequently cloned into the pEGFP-N1 (Stratagene, Santa Clara, CA, USA).

### Ahnak knock-out cell line

Stable knock-out of LGALS3BP was performed in C3H10T1/2 cells. Cells were seeded in 60 mm cell culture dishes at a density of 5 × 10^5^ cells per well. The next day, cells were transfected with Ahnak or control CRISPR/Cas9 plasmid using the TransIT-Xs Dynamic delivery system. Transfected cells were collected by Cell Sorter (Sony, Tokyo, Japan) and cloned in 96 well culture plates for 2–3 weeks for selection of Ahnak Knock-out clones.

### Semi-quantitative RT-PCR

RT-PCR analysis was performed, as described previously^36,37^. Briefly, the total RNA was harvested from treated cells using TRIzol reagent (Invitrogen, Carlsbad, CA) following the manufacturer’s protocol. One microgram of RNA was used as a template for reverse-transcription using the Prime Script 1'st strand cDNA Synthesis kit (Takara, Kyoto, Japan). PCR was performed with 20 ng of cDNA using a PCR pre-mixture (Bioneer, Deajeon, Korea). The primers used are provided in Supplementary Table 1.

### Western blotting

Western blotting analysis was performed, as described previously^36^. Protein extraction of the cells were lysed in modified RIPA buffer (1 mM Tris-HCl, pH 7.4, 100 mM NaCl, 1 mM EDTA, 1 mM NaF, 2 mM Na3VO4, protease inhibitor, 0.5 % Deoxycholate, 1 % NP-40 and 1 % Triton X-100). Equal amounts of protein (20–50 μg) were loaded in 8 % or 10 % sodium dodecyl sulfate–polyacrylamide gel electrophoresis (SDS-PAGE) and transferred by blotting to polyvinylidene fluoride membranes (PVDF; EMD Millipore, Billerica, MA, USA). Detection was performed using EZ-Western Lumi Pico (DoGen, Seoul, Korea) according to the manufacturer’s instructions.

### Immunohistochemistry and immunofluorescence

The iWAT tissues were collected, fixed with formalin, and cut into 5-μm slices. Paraffin-embedded sections of iWAT were deparaffinized, rehydrated, and subjected to immunohistochemistry. For immunohistochemistry, sections were incubated with Ahnak and Bmpr1α antibodies overnight at 4 °C. Antibody detection was performed using the EnVision detection system (Dako North America Inc., Carpinteria, CA, USA) according to the manufacturer’s protocol. For Immunofluorescence assays, cells were plated on 4-well chamber slides (Nunc, Rochester, NY, USA) and fixed in cold methanol at −20 °C for 15 min. Chamber slides were incubated overnight with specific antibodies at 4 °C. Cells were washed three times in PBS containing 0.1% Triton X-100 in PBS (PBS-T), then incubated with Alexa fluorochrome conjugated secondary for 2 h. After PBS-T washing, slides were mounted using Fluoroshield with 4′,6-diamidino-2-phenylindole (DAPI) mounting media (Immunobioscience, Mukiteo, WA, USA) and imaged by confocal microscopy (Zeiss, Oberkochen, Germany).

### Promoter assay

Transfection was performed using TransIT-Xs dynamic delivery system (Mirus) as described above. Cells at 90% confluence were transfected with pEZX-PG04 for negative control, pEZX-Gapdh, pEZX-Bmpr1α, and pEZX-Bmpr2 promoter plasmid (Genecopoeia). After 2 days, supernatant were collected and kept at −20 °C until assayed. Gaussia luciferase (GLuc) and secreted alkaline phosphatase (SEAP) were measured in supernatant using Secrete-Pair Dual Luminescence Assay Kit (Genecopoeia). SEAP activity was used for normalization such as an internal control for transfection efficiency.

### Statistical analysis

Means and S.D. were calculated using Microsoft Excel software (Microsoft, Redmond, WA, USA). A two-tailed Student’s *t*-test and unpaired Student’s *t*-test were used for statistical analysis of comparative data. Values of *p* < 0.05, *p* < 0.01, and *p* < 0.005 were considered significant depending on the experiment. Pearson correlation (*p* value < 0.05) and microarray data set (GSE28598) constructed by Yadav et al. was used to verify the co-expression patterns between Ahnak and TGF- signaling pathway gene sets.

## Electronic supplementary material

Supplementary Table S1
