# Supplementary material for: Essential role of Ahnak in adipocyte differentiation leading to the transcriptional regulation of Bmpr1α expression
Source: Cell Death Dis. 2018 Aug 28;9(9):864. doi: 10.1038/s41419-018-0873-6 (PMC6113281; doi:10.1038/s41419-018-0873-6)
Supplement: Supplementary file 1 — Supplementary Table S1 [file 41419_2018_873_MOESM1_ESM.pptx]

## Slide 1
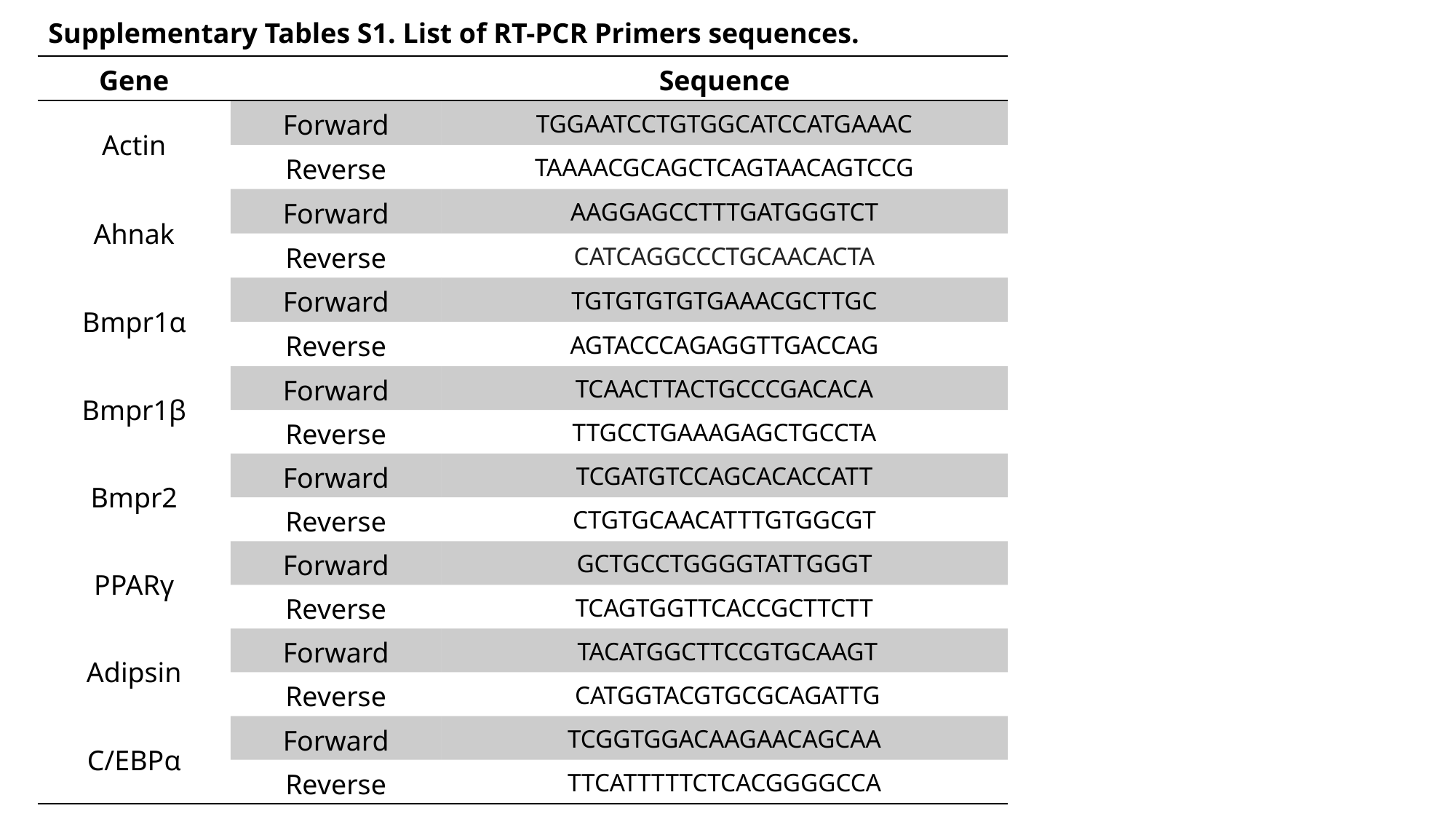

Supplementary Tables S1. List of RT-PCR Primers sequences.
| Gene | | Sequence |
| --- | --- | --- |
| Actin | Forward | TGGAATCCTGTGGCATCCATGAAAC |
| | Reverse | TAAAACGCAGCTCAGTAACAGTCCG |
| Ahnak | Forward | AAGGAGCCTTTGATGGGTCT |
| | Reverse | CATCAGGCCCTGCAACACTA |
| Bmpr1α | Forward | TGTGTGTGTGAAACGCTTGC |
| | Reverse | AGTACCCAGAGGTTGACCAG |
| Bmpr1β | Forward | TCAACTTACTGCCCGACACA |
| | Reverse | TTGCCTGAAAGAGCTGCCTA |
| Bmpr2 | Forward | TCGATGTCCAGCACACCATT |
| | Reverse | CTGTGCAACATTTGTGGCGT |
| PPARγ | Forward | GCTGCCTGGGGTATTGGGT |
| | Reverse | TCAGTGGTTCACCGCTTCTT |
| Adipsin | Forward | TACATGGCTTCCGTGCAAGT |
| | Reverse | CATGGTACGTGCGCAGATTG |
| C/EBPα | Forward | TCGGTGGACAAGAACAGCAA |
| | Reverse | TTCATTTTTCTCACGGGGCCA |
